# Oleic Acid Uptake Reveals the Rescued Enterocyte Phenotype of Colon Cancer Caco-2 by HT29-MTX Cells in Co-Culture Mode

**DOI:** 10.3390/ijms18071573

**Published:** 2017-07-20

**Authors:** Emmanuelle Berger, Merian Nassra, Claude Atgié, Pascale Plaisancié, Alain Géloën

**Affiliations:** 1 Univ-Lyon, CarMeN Laboratory, Inserm U1060, INRA U1397, Université Claud Bernard Lyon1, INSA Lyon, Fr-69100 Villeurbanne, France; pascale.plaisancie@inserm.fr (P.P.); alain.geloen@insa-lyon.fr (A.G.); 2 Institut CBMN (Chimie et Biologie des Membranes & Nanoobjets)-UMR 5248 (CNRS-Bordeaux INP-Université de Bordeaux)-Equipe ClipIN-Bât B14-Ave G. St Hilaire, 33600 Pessac, France; nassra_nassra@yahoo.com (M.N.); claude.atgie@enscbp.fr (C.A.)

**Keywords:** cancer cell lines, co-culture, fatty acids, gene datasets, signaling pathways, intestinal barrier

## Abstract

Gastrointestinal epithelium is the unique route for nutrients and for many pharmaceuticals to enter the body. The present study aimed to analyze precisely whether co-culture of two colon cancer cell lines, mucus-producing cells HT29-MTX and enterocyte-like Caco-2 cells, ameliorate differentiation into an in vitro intestinal barrier model and the signaling pathways involved. Differentiated Caco-2 cells gene datasets were compared first to intestinal or cancer phenotypes and second to signaling pathway gene datasets. Experimental validations were performed in real-time experiments, immunochemistry, and gene expression analyses on Caco-2 versus co-cultures of Caco-2 and HT29-MTX (10%) cells. Partial maintenance of cancer-cell phenotype in differentiated Caco-2 cells was confirmed and fatty acids merged as potential regulators of cancer signaling pathways. HT29-MTX cells induced morphological changes in Caco-2 cells, slightly increased their proliferation rate and profoundly modified gene transcription of phenotype markers, fatty acid receptors, intracellular transporters, and lipid droplet components as well as functional responses to oleic acid. In vitro, enterocyte phenotype was rescued partially by co-culture of cancer cells with goblet cells and completed through oleic acid interaction with signaling pathways dysregulated in cancer cells.

## 1. Introduction

In mammals, the gastrointestinal epithelium is the largest surface facing external environment. It is the unique route for nutrients and for many pharmaceuticals to enter the body. Epithelial cells allow the passage of small molecules by several mechanisms: passive transcellular passage, passive paracellular exchanges between cells, and an energy-consuming transport across the epithelium with uptake into coated vesicles (transcytosis) [1]. In vivo experiments to study intestinal physiology raise numerous questions, which indeed result from animal models, cannot be easily transposed to human and it is difficult to draw mechanistic conclusions. The availability of an in vitro model of intestinal barrier would be extremely useful to test nutrients and drugs absorption. Amongst the in vitro models of intestinal epithelium, the Caco-2 cell line has been extensively used to measure nutrient and drug transports [2,3]. Caco-2 cells derived from a human colorectal adenocarcinoma. After confluence, this cell line differentiates into enterocytes, which represent around 80–85% of the cell population of the intestinal epithelium. However, the use of Caco-2 cells is criticized for several reasons. The permeability of the monolayer to hydrophilic molecules is poor because its tightness and resembles more colonic than small intestinal tissue [4]. Caco-2 cells overexpress the P-glycoprotein that may lead to higher secretion rates and consequently lower permeability in the absorptive direction [5]. Caco-2 cells, however, appear to over-express as well as under-express certain proteins [4]. Finally, Caco-2 lack mucus production that is characteristic of intestinal mucosa, so we developed a method of co-culture using HT29-MTX, a subpopulation of HT29 human colonic adenocarcinoma cells selected for their resistance to methotrexate (MTX) [5]. Caco-2 and HT29-MTX cells represent the two major epithelial cell types, expressing as well as carriers and receptors for nutrients, macromolecules and drug transport in the same monolayer [3,6]. However, whether Caco-2 cancer cell phenotype is rescued in the presence of HT29-MTX cells remains to be clarified.

The main advantage of using HT29-MTX cells is that they produce mucus, which brings the co-culture model closer to in vivo human conditions. Previous studies have shown that different seeding ratios of absorptive (Caco-2) and goblet (HT29-MTX) cells offer the opportunity to modify the permeability of cell monolayers [1]. Furthermore, a recent study has improved the co-culture model by defining the seeding procedure [7]. Although several studies have emphasized the interest of co-culture to mimic the intestinal barrier, very little is known on the effects of co-culture on proliferation and gene expressions [8,9,10,11,12] and its role in conversion from carcinogenic to healthy phenotype remains to be fully elucidated.

The goal of the present study was to analyze in real-time experiments the effect of co-cultures on healthy enterocytes rescued from cancer phenotype. Bio-informatic analyses of signaling pathways involved in intestinal phenotype alterations in Caco-2 colon cancer cell line pointed out a potential role of fatty acids in phenotype regulation. Thus, dose-dependent effects of oleic acid were analyzed on both cultures and co-cultures of Caco-2 and HT29-MTX cells, clearly demonstrating reprogramming of Caco-2 cell phenotype during co-culture.

## 2. Results

### 2.1. Bio-Informatic Dataset Analyses Point out the Role of Fatty Acids in Both Cancer and Intestinal Phenotypes

We previously developed a comparative analysis of gene dataset enrichment in order to identify signaling pathways significantly enriched in selected phenotypes. For example, we identified pathways related to adiponectin signaling in cancer cells [13], cell proliferation, and/or survival as well as the pathways involved in glucose signaling and specifically dysregulated in hepatocellular carcinoma [14,15]. Several of the merged pathways have been further experimentally validated, and most of them correspond to previously published data. In the present study, human datasets were selected to represent intestinal phenotype of either enterocytes or colonocytes, genes induced in differentiated human cell lines Caco-2 and HT29 cells and dysregulated in cancer cells (Table 1).

The analysis of gene datasets representing normal intestinal phenotypes revealed a list of 86 genes which are specific of mature enterocytes versus colonocytes (Table S1) and one noticeable differentially expressed gene is the thrombospondin receptor *CD36* (i.e., *FAT/CD36* fatty acid translocase). Less than 2% of the genes representing differentiated Caco-2 cell lines genes were commonly found in normal intestinal cells, such as epidermal growth factor (*EGF*) and transforming growth factor alpha (*TFNA*). It should be noted that two cancer markers, *CD44* and Bone Morphogenic Protein 4 (*BMP4*), were differentially found in the three sets of Caco-2 differentiated datasets and absent in normal cells.

**Figure 1:**
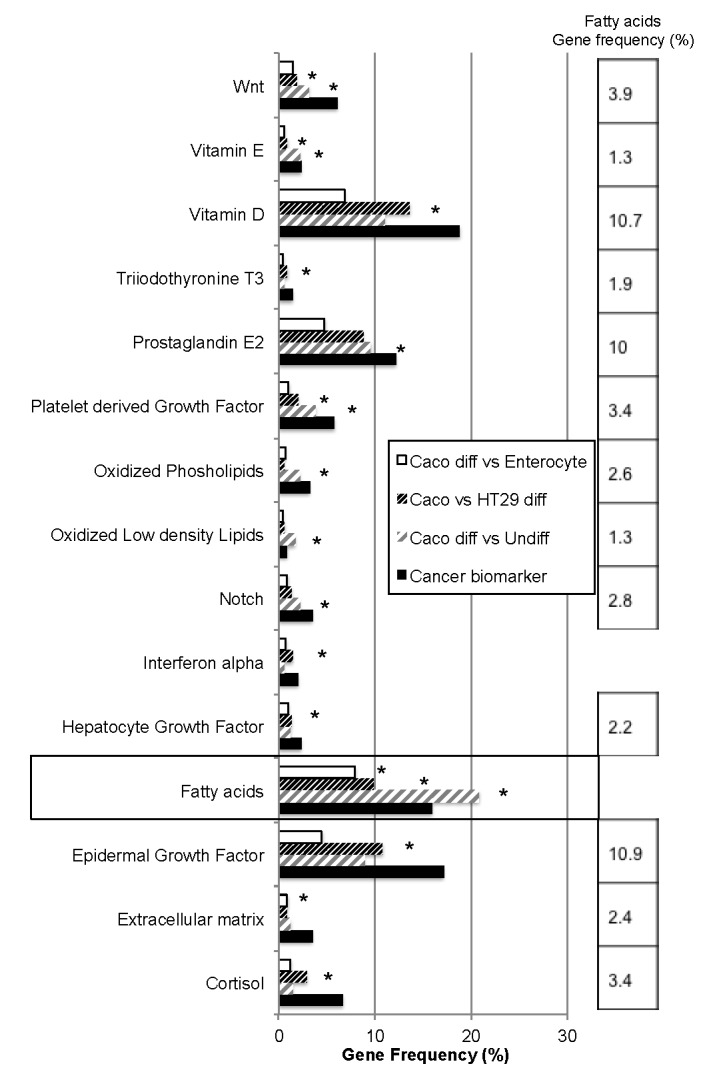
External stimulus gene datasets linked to cancer and intestinal phenotypes. Among the gene datasets regulatable by 49 different stimulus pathways analyzed, 15 are significantly over-represented in cancer phenotype and present significant enrichment in transcriptional targets with healthy intestinal and/or Caco-2 and HT29-MTX differentiated cell lines. Among them, fatty acids have significant number of common regulatable genes with all the pathways dysregulated, except Interferon alpha, their representativity in these pathways is reported in the right column. Z-score confidence level >90% versus genome are indicated by asterisks.

In the phenotype datasets 61% of the genes are under the control of at least one of the 49 extracellular signaling pathways analyzed (Table 1). Due to a low number of genes, the representativity of signaling pathways could not be applied to colonocyte nor enterocyte datasets, thus was analyzed in Caco-2 and HT29 datasets. Only 19 signaling pathways were significantly over-represented in intestinal cell lines. Except Interleukin 6 and 8, and retinol binding protein (*RBP*) pathways (over-represented in Caco-2 cells differentiated versus undifferentiated) and potassium (over-represented in Caco-2 cells versus enterocytes) all of these signaling pathways were also significantly over-represented in the cancer biomarker dataset (Figure 1).

**Figure 2:**
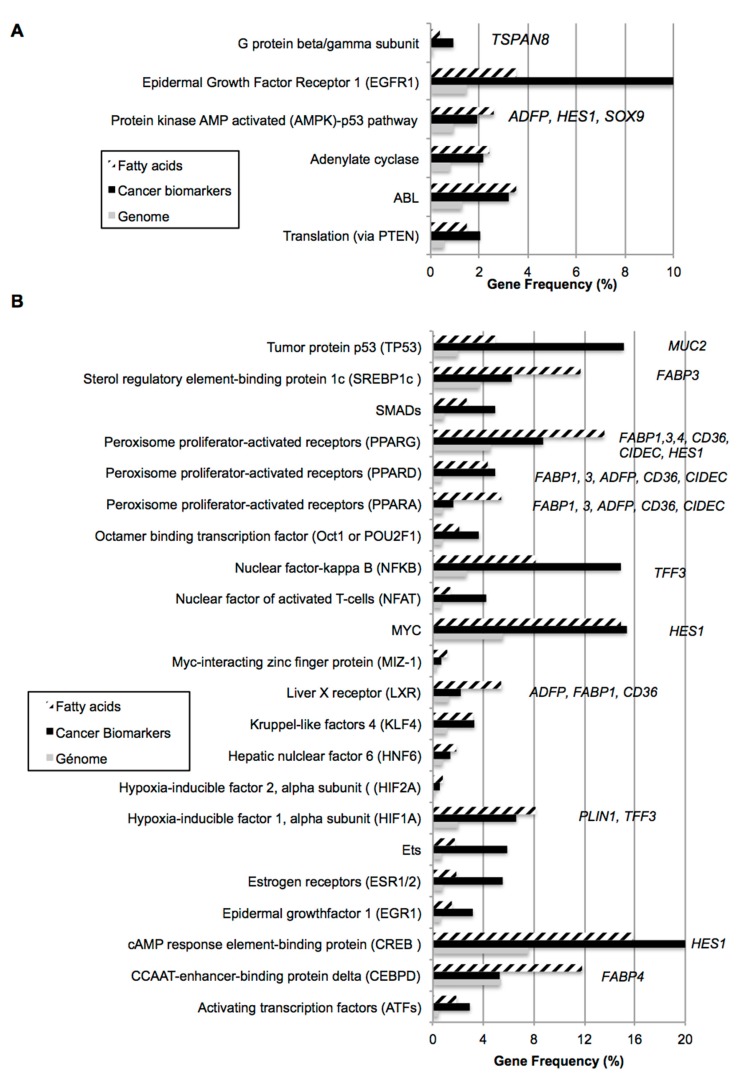
Fatty acids-linked signaling pathways related to cancer. Fatty acids gene datasets were compared to 48 gene datasets related to intracellular pathways (**A**) and 52 gene datasets related to transcription factors (**B**). Only significantly over-represented pathways in both fatty acids and cancer biomarkers datasets are represented (Z-score confidence level >90% versus genome). Target genes further analyzed by qRT-PCR are reported in italic characters.

Numerous growth factors are significantly over-represented, as well as extracellular matrix or vitamins. The most surprisingly emerging signal is fatty acid pathway, which is significantly over-represented in the three gene datasets, suggesting a major function in enterocyte. Moreover, except for interferon alpha, all the signaling pathways significantly over-represented additionally present significant crosstalk with fatty acid signaling, as reported in Figure 1. The list of genes regulatable by fatty acids in intestinal phenotype is available in Table S2. Among them, several are involved in lipid transport, uptake (such as *FAT/CD36*, low density lipid receptor *LDLR*, and fatty acid binding proteins 1–5, *FABPs*), storage (perilipin-2, *ADFP*), secretion (apoproteins *APOA2* and *APOC3*), or metabolism (such as diacylglycerol transferase 2 *DGAT2*, lipid protein lipase *LIPE*, fatty acid synthase *FASN*). Most of them, such as *FABPs* and *FASN*, are themselves regulated by fatty acids. An analysis of fatty acid-related signaling pathways was performed by comparison with 48 intracellular pathways and 52 transcription factors target gene datasets (Figure 2A,B, respectively). Only six pathways commonly over-represented in both fatty acids and cancer biomarkers datasets were identified, including adenylate cyclase and G protein, protein kinase AMP-activated kinase (AMPK)/P53, and EGF receptor pathways. Among the genes involved in intestinal phenotype, AMPK regulates hairy and enhancer of split 1 (*HES1*), trefoil factor 3 (*TFF3*), and the lipid-droplet associated protein *ADFP*. As much as 22 transcription factors were commonly significantly enriched in both fatty acids and cancer biomarkers datasets, such as metabolic responsive peroxisome proliferator-activating factors (PPARs) or well-known cancer-related factors such as V-Myc Avian Myelocytomatosis Viral Oncogene Homolog (*MYC*), which regulates *HES1*, *P53*, which regulates mucin 2 (*MUC2*), or Smads. PPARs also regulate *HES1* and numerous proteins involved in fatty acid signaling and/or storage, such as fatty acid binding proteins *FABP1*, -*3*, and -*4*, *FAT/CD36*, or lipid-associated proteins *ADFP* and cell death-inducing DFFA-like effector c (*CIDEC*).

### 2.2. Real-Time Analysis Reveals the Modulatory Activity of HT29-MTX Cells on Caco-2 Cell Growth and Differentiation

**Figure 3:**
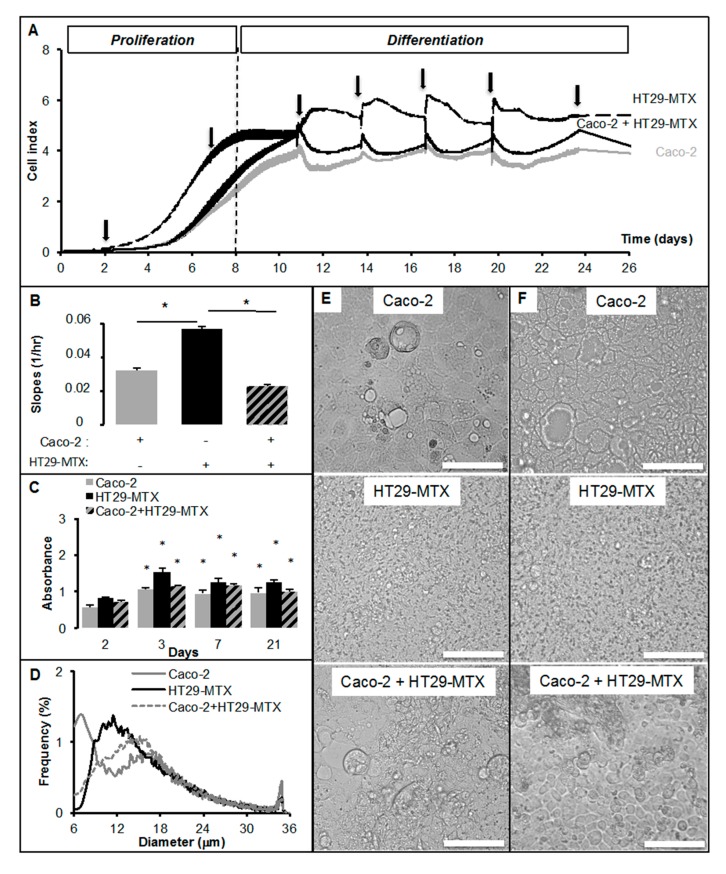
Real-time monitoring of proliferation and differentiation of Caco-2, HT29-MTX cells, and co-cultures (representative experiment). Cells were plated in either 96E-plates or 96 wells plates at low density (5000 cells/cm^2^, (**A**,**B**,**D**,**E**,**F**) or 75,000 cells/cm^2^ (**C**) and cultured separately or mixed (Caco-2 cells: 90%; HT29-MTX cells: 10%). The cell indexes were measured on xCELLigence system (**A**) every 15 min and the media were replaced every 2–4 days (indicated by arrows) until full differentiation, i.e., 18 days after they reached confluency (Day 8); (**B**) Proliferating phase slopes were calculated from the linear phase. Data are presented as means ± SEM (*n* = 8 wells); (**C**) Cell survival and/or proliferation was checked using MTT test; (**D**) Cell size distribution at Day 18 was analyzed on Scepter cell counter. Data are presented as mean ± SD (*n* = 3); Micrographs taken 2 days (Day 2, **E**) and 18 days (Day 18, **F**) post-confluency at magnification ×20 (scale bar = 100 μm). Asterisks represent significant Student *t*-test *p*-values < 0.05.

In the next step, we analyzed in real-time experiments Caco-2 and HT29-MTX cell cultures separately or in co-cultures (ratio 9/1) onto xCELLigence system, which cell index reflects cell surface occupancy, i.e., takes into account cell size, cell number, and adhesion force (Figure 3A).

**Figure 4:**
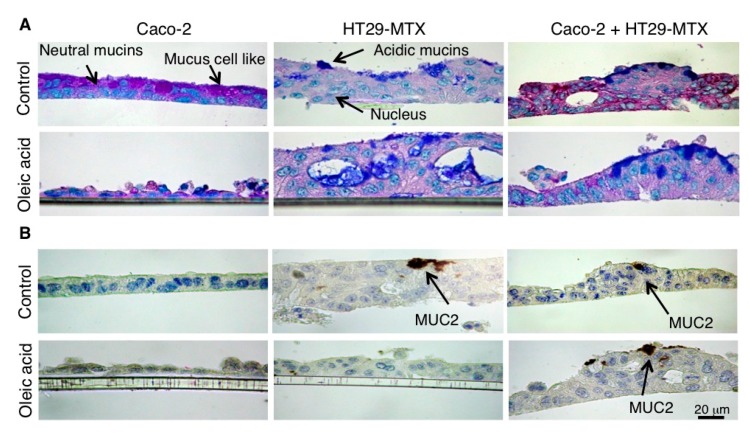
Morphological changes induced in Caco-2 and HT29-MTX cells in co-cultures and the effects of oleic acid. Caco-2 and HT29-MTX cells were cultured separately or mixed (9/1) until full differentiation (Day 18) on 12-well insert plates and treated for 4 h with either vehicle (Control) or oleic acid complexed to bovine serum albumin (20 μM) then fixed with Carnoy’s reagent for detection of (**A**) Periodic acid Schiff (PAS) and Alcian blue (AB) staining, acidic mucin (blue) and neutral and sialylied mucins (pink magenta) and (**B**) mucin 2 (MUC2) immuno-histochemical detection (Brown-black), with FastRed/naphtol detection of phosphatase alcalin (PA) activity (red) and Mayer hematoxilin labeling of tissue.

When plated at low density, the slope of the linear phase of cell index (4–5 days after plating) represents the rate of proliferation (Figure 3B). Both delta cell indexes (Figure 3B) and MTT test (Figure 3C) suggest that HT29-MTX cell proliferation rate was higher than that of Caco-2 cells. In Caco-2/HT29-MTX co-cultures (Ratio 9/1), the mean slope was 0.034 (doubling time about 36 h) instead of 0.023 (doubling time around 46 h for Caco-2 cells alone). This result reveals that as little as 10% of HT29-MTX cells may slightly modulate the rate of Caco-2 cell proliferation. Confluence was reached earlier (8 days after plating) in co-culture than in separated cultures, suggesting additional effects on cell morphology. HT29-MTX cells are smaller than Caco-2 cells (modes 11.99 μm for HT29-MTX cells, 15.63 μm for Caco-2 cells, and 15.13 μm in co-cultures (Figure 3D). At confluence (cell index at 4.5) media replacements also revealed differential effects on cell cultures, with a slight reduction of cell index in both HT29-MTX and Caco-2 cells, although a slight increase of the cell index was observed after media replacement in co-culture. These slight effects may reveal differences in metabolic activities according to the modification of media activities with time (nutriment management, secretory activity of cultured cells). In order to evaluate the morphological changes induced by co-culture, Caco-2, HT29-MTX, and Caco-2/HT29-MTX cells were grown on membrane filters and examined 18 days post-confluence using histochemicals and immuno-histochemicals (Figure 4).

Caco-2 cells appeared slightly stratified and cuboidal or as a simple columnar monolayer of polarized epithelial cells with some mucus-like cells. However, no mucin 2 (MUC2) immunoreactivity was detected in this cell line. On 18 days post-confluence, HT29-MTX cells rarely appeared as a simple monolayer but are closer to a stratified epithelium. In this cell line, the intracellular mucus granules were stained in blue by Alcian Bule (AB)/Periodic acid Schiff (PAS) and MUC2 immunoreactivity was detected in some goblet-like cells as shown in Figure 4A, the co-culture formed a stratified epithelium with numerous small cells. These cells were highly PAS positive. The HT29-MTX cells appeared as islets of cells and were readily identifiable by the AB staining.

### 2.3. Quantitative RT-PCR Analyses

Gene transcription of phenotype markers was analyzed by qRT-PCR of fully differentiated cultures (Day 20, Table 2). In HT29-MTX cells the expression of *HES1* and *ATOH1* (*Hath1*) noticeably decreased. *Gfi1*, a transcription factor implicated in goblet cell differentiation, was also decreased in the co-culture as compared with the expected values calculated from 90% mRNA from Caco-2 cells + 10% mRNA from HT29-MTX cells. In contrast, *Sox9* mRNA expression level was not significantly modified in co-culture. *MUC2* was preferentially transcribed in Caco-2 cells although *MUC5AC* was highly expressed in HT29-MTX cells. The intestinal regulator *TFF3* was highly expressed in both cell lines. In co-cultures, the presence of HT29-MTX cells drastically reduced the transcription of these three goblet cell markers, 6 to 8 fold the expected values calculated from 90% mRNA from Caco-2 cells + 10% mRNA from HT29-MTX cells.

We also analyzed the basal transcriptional levels of fatty acid receptors, intracellular transporters and of genes involved in lipid droplet structure and function (Table 2). The major fatty acid receptor transcribed in both cell lines is the G-protein coupled receptor *GPR120*, in comparison to a poor transcription of the fatty acid translocase *FAT/CD36*. The fatty acid binding proteins (FABPs) were highly transcribed in Caco-2 cells, with a predominant detection of *FABP1*. Among mRNAs coding for the lipid droplet associated proteins, Abhydrolase Domain Containing 5 (*ABDH5*), perilipin 3 (*PLIN3*), and Cell Death-Inducing DFFA-like Effector C (*CIDEC*) were highly expressed in Caco-2 cells. All lipid droplet associated proteins analyzed were more abundant in Caco-2 cells than in the co-culture. Finally, the cancer marker tetraspanin 8 (*TSPAN8*), which transcript was highly detected in both cell lines, was strongly reduced in co-cultures. All of these genes were strongly reduced in co-cultures, without any significant effect on the stress-responsive gene *XBP1*. In conclusion, the presence of 10% HT29-MTX in Caco-2 differentiated cells induced a strong transcriptional down-regulation of genes involved in either intestinal cell phenotype and lipid uptake.

### 2.4. Real-Time Uptake of Oleic Acid by Caco-2, HT29-MTX Cells, and Co-Cultures

**Figure 5:**
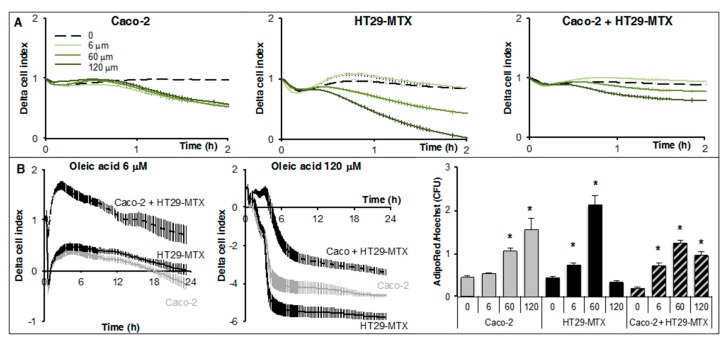
Real-time monitoring of lipid uptake by Caco-2, HT29-MTX cells, and in co-cultures (9/1). Real-time cell analyses (RTCA) were performed on fully differentiated cells (Day 18) treated with several doses of oleic acid complexed to bovine serum albumin. These are representative experiments presented as mean values ± SEM (*n* = 8 wells) with significant Student *t*-test *p*-values <0.05) represented by stars. (**A**) xCELLigence analysis of delta cell index (cell indexes normalized at time of treatment) on a short term experiment (2.5 h) in presence of increasing concentrations of oleic acid; (**B**) Comparative analysis on xCELLigence of cells treated by oleic acid 6 (left panel) or 120 μM (central panel) for 24 h then fixed for triglyceride storage measurement (AdipoRed intensity) normalized to nuclei counts (Hoechst counts) on Cytation 3 plateform (right panel). Cell indexes were measured every 2 min for 1.5 h then every 15 min.

In a next step, the ability to uptake oleic acid by Caco-2, HT29-MTX cells, and co-cultures was monitored using real-time measurements. In a previous study, we described a new method to measure lipid uptake on xCELLigence system in short-term experiments (few hours) by measuring the cell adhesion force modified by lipid storage [16]. Fully differentiated cells plated onto 96E-plates were treated by several doses of oleic acid complexed to bovine serum albumin in serum-free media and low glucose concentrations (Figure 5A).

**Figure 6:**
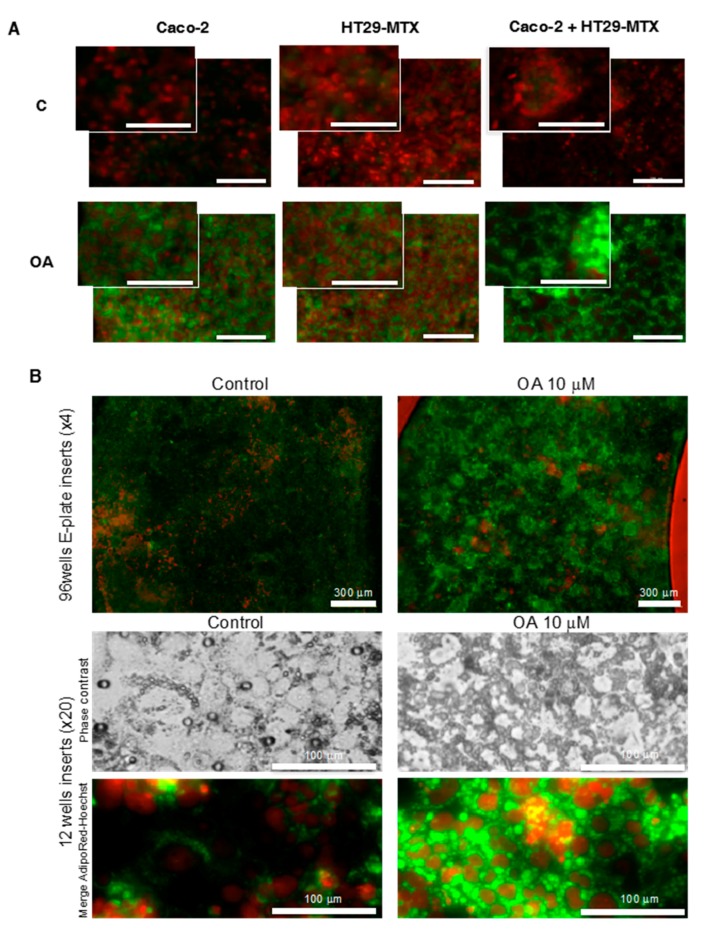
Morphological changes in lipid storage in Caco-2, HT29-MTX cells, and co-cultures. Cells were treated with oleic acid 10 μM for 4 h then with AdipoRed and fixed with formalin 10% before staining with Hoechst 33258. Micrographs were acquired with identical parameters at either ×4 and ×20 magnification on Cytation 3 plateform separately and are presented as merged images of AdipoRed-labeled lipid droplets (green) and Hoechst-labeled nuclei (red). (**A**) Plated Caco-2, HT29-MTX cells, and co-cultures (scale bar = 100 μm); (**B**) Insert Caco-2/HT29-MTX co-cultures treated without or in presence of oleic acid at 10 μM for 4 h.

A significant reduction of cell index was observed in both Caco-2 and HT29-MTX cells with a much higher effect on HT29-MTX cells. The decrease in cell index was the lowest in the co-culture (Caco-2 + HT29-MTX) in comparison to that of single cell lines. No dose response effect was observed for Caco-2 cells. Real-time Cell Index Analyzer (RTCA) cell index takes into account both cell adhesion and cell number. Therefore, further analyses of lipid accumulation and cell count were performed using fluorescence imaging and automated counting of fatty acid storage as triglycerides and nuclei number, respectively. High doses of oleic acid induced cell death in non-adipose cells depending on cell culture; therefore, dose-dependent analyses are required in each experiment. In a representative experiment, dose-dependent responses from 6 to 120 μM oleic acid showed that HT29-MTX are more sensitive than Caco-2 cells (Figure 5A,B) and that the highest dose might have deleterious effect on their survival. Thus, lower doses ranging from 0.5 to 20 μM were applied in order to avoid such a toxic effect in further experiments. Nuclei counts were performed as controls and for normalization of TG storage to cell number. A dose-dependent accumulation of triglycerides was induced by oleic acid in either Caco-2, HT29-MTX cells, or co-cultures (Figure 5B). Surprisingly, fluorescence imaging of fatty acid storage revealed a drastic remodeling of diffuse lipid droplets in both Caco-2 and HT29-MTX cultures into lipid-droplet like structures in co-cultures (Figure 6).

### 2.5. Transcriptional Regulations by Oleic Acid on Caco-2, HT29-MTX Cells and Co-Cultures

**Figure 7:**
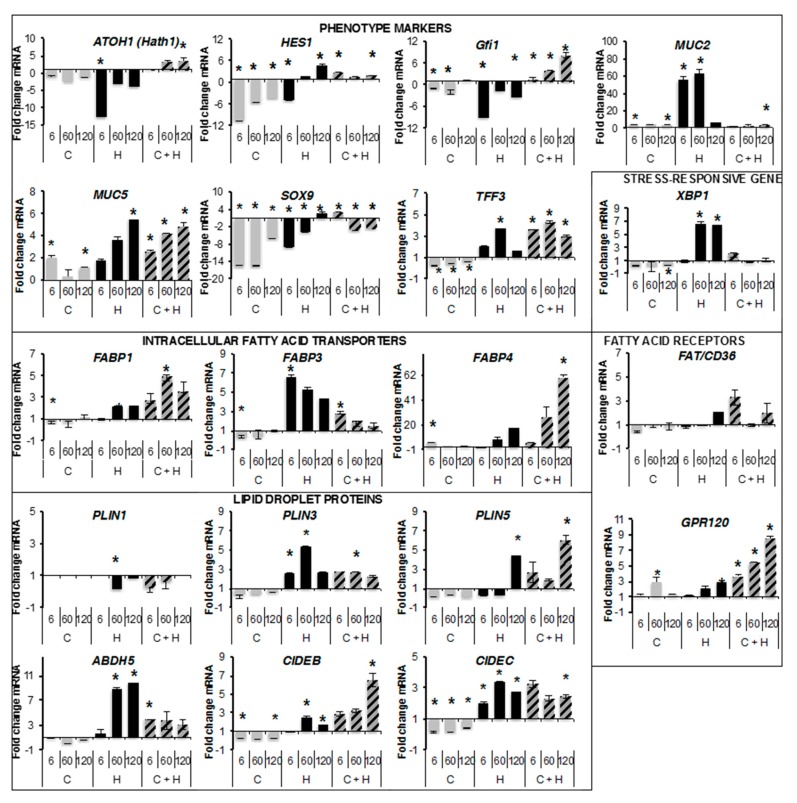
Transcriptional regulations by oleic acid in Caco-2 (C), HT29-MTX (H) and co-cultures (C + H). Fully differentiated cultures (Day 18) were treated for 48 h with several doses of oleic acid complexed to bovine serum albumin (representative experiment). mRNA quantifications are presented as fold changes of treated versus control samples normalized to those of HPRT and measured in triplicates on cumulated 8 wells from 96-wells plates. Data are presented as mean values ± SD and significant Student *t*-test *p*-values < 0.05 are indicated by stars.

The transcriptional effects of oleic acid onto phenotype markers and the proteins involved in fatty acid uptake and storage were monitored on fully differentiated cells (Day 18) treated for 48 h with different doses of oleic acid (Figure 7).

The highest dose 120 μM may induce a stress response by increasing *XBP1* mRNA levels in HT29-MTX, suggesting that it is a toxic dose for these cells. It did not affect Caco-2 cells nor co-culture, according to low nuclei number counts in inserts (Figure 5B). Oleic acid reduced the transcription of *MUC2* into Caco-2 cells although it was increased in HT29-MTX cells for the two lowest concentrations of oleic acid. *MUC5AC* transcription was induced in either Caco-2, HT29-MTX cells, or co-cultures by oleic acid. That of *TFF3* which was also reduced in Caco-2 cells, increased in HT29-MTX cells at 60 μM oleic acid and was induced in co-culture. These data indicate that oleic acid modulates the phenotype of these cell lines in both independent cultures and in co-culture. *FAT/CD36* transcription remained unchanged by oleic acid while that of *GPR120* was strongly increased in a dose-dependent manner in co-cultures. The transcript abundance of fatty acid transporters were mainly inhibited or unchanged by oleic acid in Caco-2 cells, only *FABP3* transcription was increased in HT29-MTX cells in response to oleic acid, while in co-culture oleic acid stimulated the *FABP1*, *3* and *4* at different concentrations. The lipid droplet-associated proteins were regulated at the transcriptional level by oleic acid, mostly reduced in Caco-2 cells, increased in HT29-MTX cells and in co-culture (except *PLIN1*). This was particularly noticeable for the most abundant transcripts for *GPR120*, *FABP1*, *PLIN3*, and *CIDEC*. In summary, most of the proteins associated with fatty acid uptake and storage were found regulated by oleic acid at the transcriptional level, this was particularly noticeable for the most abundant transcripts for *GPR120*, *FABP1*, *PLIN3*, and *CIDEC*. In conclusion, the transcriptional induction of either phenotype markers (except for *TFF3*), and lipid uptake and/or storage in co-cultures suggests that non-toxic doses of oleic acid (5 μM) may increase enterocyte phenotype.

## 3. Discussion

Both Caco-2 and HT29-MTX cell lines originate from human colon cancer, respectively from enterocytes and goblet, i.e., mucus-producing cells. Several models of cultures and co-cultures suggest that three weeks of maintenance at confluence induces functional intestinal barrier reconstruction [15]. However, the Caco-2 cell phenotype has been reported to be only partly rescued from cancer phenotype, i.e., partial conversion of colonocytes to enterocyte phenotype through coordinated down-regulation of genes involved in cell proliferation and up-regulation of genes involved in xenobiotic and drug metabolism, lipid transport, and metabolism, brush border and junction proteins [15]. Although numerous studies have shown the major role of the extracellular matrix, the exact impulses required for full differentiation into highly polarized cells remain largely unknown [17]. Using comparative analysis of common gene transcriptional regulations by a series of human external signals and experimental validations, we provided strong evidence of a potential role of fatty acids and at least oleic acid in such a process.

Using three different gene datasets representative of differentiated Caco-2 cells, we found the remaining of well-described colon cancer phenotype markers CD44 [18] and BMP4 [19] in accordance with a poor healthy phenotype restoration. This result is in accordance with the cancer status of Caco-2 cells, which carry mutation in several genes such as *P53*, APC, WNT signaling pathway regulator *APC*, beta catenin, and Smads [20]. Among extracellular signals pathways over-represented in differentiated Caco-2 cells most of them are well described in the process of colon cancer, such as Notch, extracellular matrix, Wnt [21,22,23,24], or EGF, which is involved in differentiation [25]. The present results show that the introduction of a small proportion of goblet-like cells during the Caco-2 cell culture significantly modulates cell growth and differentiation. The initial rate of cell proliferation was very different between Caco-2 cells and HT29-MTX, the later showing a higher rate of proliferation. Consequently, confluence was reached much faster in HT29-MTX cells than in Caco-2 cells. The addition of 10% of HT29-MTX to Caco-2 cells significantly modified the rate of cell proliferation (Figure 2). Interestingly, it also increased the value of the cell index at confluence. During differentiation, the cell index remained higher in the co-culture than in Caco-2 cells. Since the cell index takes into account not only the cell number but also cell size and adhesion force, that result suggests a significant qualitative difference in the co-culture. Indeed, in the co-culture of Caco-2 cells (90%)/HT29-MTX (10%), we observed a down-regulated transcription of transcription factors important in cell fate determination (*HES1*, *ATOH1*, *GFi1*, *Sox9*) and of specific markers of epithelial differentiation (*MUC2*, *MUC5AC*, *TFF3*) when compared to Caco-2 cells or HT29-MTX alone. The intestinal epithelium is characterized by a fast and constant renewal occurring every 3–5 days. To achieve this, the multipotent stem cells divide and produce an actively proliferating population of transit amplifying cells that differentiate during a series of steps into either absorptive enterocytes, the predominant population, or the secretory lineages. Several experiments have demonstrated that the Notch cascade plays a critical role in cell fate decision between the absorptive and secretory lineages [26]. When the Notch pathway is activated, its downstream target gene, *HES1*, represses the expression of the helix-loop-helix (HLH) protein *Hath1* (*ATOH1*) and promotes an absorptive cell fate over a secretory cell fate. In contrast, Hath1 is essential for the production of intestinal secretory cells (goblet, enteroendocrine, and Paneth cells). The differentiation and maturation of goblet and Paneth cells are then controlled by several genes such as *Gfi1*, SAM pointed domain containing ETS transcription factor *SPDEF*, and Kruppel like factor 4 *Klf4* [27]. Surprisingly, our study showed that the introduction a small proportion of HT29-MTX (goblet-like cells) during the culture of Caco-2 cells (enterocyte-like cell) drastically decreased the transcription of *HES1* and *ATOH1*. These data could suggest a decline of the epithelial differentiation. In agreement with this hypothesis, the mRNA levels of goblet cell phenotype markers such as *MUC2* were strongly decreased in the Caco-2 cells/HT29-MTX co-culture. More surprisingly, fatty acid uptake and transport or lipid droplet genes were also drastically reduced.

The morphological analysis also revealed that, while Caco-2 cells tend to form a monolayer of cuboid cells after confluence, the Caco-2 cells in the co-culture took the form of a multilayered of small cells with no evidence of differentiation (containing neutral glycoproteins). In this context, the decreased transcription of *TSPAN8* observed in the co-culture was more surprising. Indeed, TSPAN8, a member of the tetraspanin superfamily, is greatly overexpressed in several types of cancer, including colorectal, liver, pancreatic, and gastric cancers and this overexpression correlates with a poor differentiation. Le Naour et al. [28] have shown that TSPAN8, also named Co-029, could be highly expressed in epithelial cells in normal colon, opposite to a low expression level in tumors. Therefore, heterogeneity in terms of expression level was observed on metastasis. Together, these data suggest that, under our conditions of cell culture, as little as 10% of HT29-MTX (goblet-like) cells, which derived from an aggressive colon cancer cell line, have the ability to alter the differentiation of Caco-2 cells.

Among the external signals identified as potential regulators of Caco-2 cell differentiation, gene dataset analyses revealed a significant over-representativity of fatty acids pathway commonly found in the three phenotypes (Figure 1). Moreover, the transcriptional activity significantly cross-reacts with all other identified pathways (except that of interferon alpha), suggesting that they may constitute a major impulse required for full differentiation. Such a hypothesis has been previously pointed out. Colonocyte differentiation status has been shown to dictate its lipid composition in healthy epithelium through gradients of lipid enzymes expression, characterized by altered expression in colon cancers [29]. Moreover, the reduction of palmitoleic and oleic acid accumulation as phospholipids is significant in colon cancer tissues and is strongly linked to cancer progression [30]. Thus, we used dose-dependent responses to oleic acid in order to study its impact on both Caco-2 cells alone and in co-culture with HT29-MTX cells at the level of phenotype and transcriptional activities. We observed that oleic acid exerted differential effects on Caco-2 cells, depending if they were grown alone or in the presence of HT29-MTX cells (10%). Bio-informatic analyses suggest that free fatty acids may play an important role in intestinal phenotype through transcriptional regulation of several genes involved in fatty acid signaling, uptake and metabolism (Figure 1 and Table S2). Indeed, oleic acid increased the mRNA levels of fatty acid receptors, intracellular fatty acid transporters, and lipid droplet components in the co-culture, whereas it had either no effect or a repressive effect on the transcription of these factors on Caco-2 cells cultured alone. For example, oleic acid stimulation increased the mRNA level of *GPR120* (an intestinal sensor of fatty acids) and of *PLIN3* (also named *TIP47*) in the co-culture but not in Caco-2 cells. Since the expression of PLIN3 in intestinal cells is known to be higher after an acute high-fat challenge [31], the co-culture gives a more “physiological” response than the single cell line. We also noted that, when tested in Caco-2 cells alone, oleic acid altered the morphology as well as the transcription level of *FABP1*, *FABP3*, *CIDEB*, and *CIDEC*. The histological analysis showed in particular that the Caco-2 cells have lost their cuboid morphology after stimulation with 20 μM oleic acid. In contrast, after stimulation with oleic acid, HT29-MTX cells have strongly increased transcripts of the three markers of goblet cells (*MUC2*, *MUC5AC*, and *TFF3*), suggesting that this fatty acid enhances the goblet cell differentiation. A very strong increase of *XBP1* mRNA levels was also induced in these cells upon stimulation by oleic acid. Indeed, we found that HT29-MTX cells were more sensitive than Caco-2 cells to oleic acid in dose-dependent experiments (Figure 5). This could indicate that high doses of this fatty acid induced a stress response and was toxic for these cells. However, a recent study clearly demonstrated that XBP1 is essential for intestinal goblet cells and that it modulates their number and their size as well as the expression of *MUC2* [32]. The amplified transcription of *XBP1* observed in our study could therefore also be indicative of an increased mucipare differentiation.

Bio-informatic analyses lead to identify signaling pathways commonly regulated by fatty acids and dysregulated in cancer cells (Figure 2). For example, fatty acid signaling is enriched in P53 target genes, possibly through AMPK activation. AMPK is a well known target of fatty acids, which in turn activates reactive oxygene species (ROS) production and caspase-3 activity [33]. This pathway could participate in the phenotype reprogramming observed in Caco-2/HT29MTX co-cultures treated with oleic acid through transcriptional induction of *HES1*, since this gene is inducible by AMPK in cancer cells [13]. Oleic acid also inhibits stress-operated calcium entry, which in turn reduces cell proliferation of colon cancer cells [34]. This could be reliable to over-representativity of the calcium-sensitive Nuclear Factor Activated in T-cells, NFAT pathway, in fatty acid regulatable gene dataset (Figure 2B). Other fatty acid-regulated transcription factors such as PPARs may also be involved in fatty acid signaling as both PPAR beta and gamma activations inhibit colon carcinogenesis [35,36]. In gene datasets, genes involved in lipid signaling *FABP1*, -*3*, and -*4*, *FAT*/*CD36*, *ADFP*, and *CIDEC* are also regulatable by PPARs and further validation would be required. PPAR alpha regulates the transcription of many intestinal barrier genes as well as genes involved in beta oxidation [37] and the transcription of fatty acid receptor *FAT/CD36* itself is regulated by PPAR alpha and gamma [38]. Whether the PPAR alpha activity on transcriptional regulation by free fatty acids acts through LKB1/AMPK and/or production of ROS is not clear [39] and remains to be further investigated in intestinal cells. In a previous study, we showed transcriptional induction of *CIDEA*, *CIDEC*, *FABP4*, and *FAT/CD36* itself by oleic acid through FAT/CD36 [16], suggesting the FAT/CD36 pathway may participate in oleic-acid mediated intestinal cell reprogramming. We also validated that both extracellular FAT/CD36 and FABP4 proteins were reduced in co-cultures (Figure S1). This hypothesis is reinforced by previous studies showing that FAT/CD36 plays an important function in intestinal differentiation and fatty acid release through cAMP, protein kinase A, and ERK1/2 signaling [40,41,42]. Although oleic acid-induced activity (at least in early events of fatty acid uptake) and the functional implication of FAT/CD36 in the processing of chylomicrons have been clearly established, the mechanism involved in cell reprogramming requires further analyses.

## 4. Materials and Methods

### 4.1. Bio-Informatic Dataset Enrichment Analyses

In previous studies, we described a novel approach using large-scale gene transcription analyses, mainly microarray dataset analyses, to explore significant enrichment in a given gene dataset, i.e., related to selected phenotype, of specific signaling pathways regulated by either external stimulus, intracellular pathways or transcription factors [13,14,17]. Briefly, each dataset was compared to human gene lists under the control of a given signaling pathway. In the present study, we selected datasets representative of several human intestinal phenotypes (Table 1), which were compared to 49 different external stimulus responsive gene datasets. Fatty acids and cancer biomarker datasets was compared to 48 intracellular pathways and 52 transcription factors pathways. Significance was calculated using z-score confidence level >90% in comparison to the genome.

### 4.2. Cell Culture

Caco-2 cells are enterocyte-like cells originating from human colorectal carcinoma (ATCC, Molsheim, France) and HT29-MTX cells, a human colon carcinoma-derived mucin-secreting goblet cell line, were provided by T. Lesuffleur (INSERM U560, Lille, France). Both cell lines were grown separately or in co-cultures (Caco-2/HT29-MTX 9/1) in Dulbecco’s Modified Eagle’s Medium (DMEM) with high glucose (4.5 g/L) containing 10% fetal calf serum (DMEM 10% FCS, PAA Laboratories, Les Mureaux, France) and antibiotics (streptomycin 100 μg/mL and penicillin 100 units/mL, Sigma Aldrich, St. Quentin Fallavier, France). Differentiation was completed 18 days post-confluency.

### 4.3. Real-Time Cell Analysis

The method has been extensively described in a previous study [16]. Briefly, cells were seeded at high density (20,000 cells/cm^2^) for differentiation experiments or 5000 cells/cm^2^ for proliferation assays in either E-plates 96, E-view 96, Costar 96-wells, or 12 wells plates for complementary analyses. Cells were grown at 37 °C in 5% CO_2_ in DMEM, 10% FCS with high glucose, and removed using trypsin 0.05% (PAA Laboratories, Les Mureaux, France). Cell proliferation, survival, and differentiation were monitored with the xCELLigence Real-time Cell Analyzer (RTCA) System (ACEA Biosciences, Inc., San Diego, CA, USA), which allows label-free monitoring changes of cell number, viability, morphology, and quality of cell attachment by measuring cell-to-electrode responses in E96-well plates manufactured with integrated microelectronic sensor arrays. RTCA system measures cell surface occupancy, i.e., cell index, taking into account cell number, cell size, and adhesion force. These three cumulative parameters were distinguished using complementary analyzes of cell size, number, and storage of triglycerides. The results are represented as cell indexes or delta cell index (i.e., (cell index at selected time minus cell index at time of treatment) divided by cell index at time of treatment). Thus, the delta cell index represents the modification of cell index at time t versus cell index at time of treatment. It was applied on non-linear RTCA profiles. Proliferation rates (slopes of linear proliferative phases) and cell indexes may vary from an experiment to another, for that reason data are representative experiments of at least three independent experiments and each condition was tested in 8 replicates. Data are presented as mean values ± SEM.

### 4.4. Cell Survival and Size Analysis

Cell survival during proliferative and differentiating phases were checked on 75,000 cells/cm^2^ plated onto 96-wells plates using MTT test standard protocol (Sigma Aldrich). Scepter handled cell counter (Merck Millipore, S.A.S., St Quentin-en-Yvelines, France) was used with 60 μm tips on living cells in suspension both for quality control of cell plating and cell size distribution analyses, measured at least in triplicates. Results are presented as mean values ± SD with significant differences for Student’s *t*-test *p*-values < 0.05.

### 4.5. Treatments

Oleic acid (Sigma Aldrich) was diluted at 200 μM in lipid-free bovine serum albumin (BSA) 5%, pre-incubated at least 2 h at 37 °C before use then filtered. Dilutions were performed in serum-free DMEM with glucose 4.5 g/L culture media.

### 4.6. Histochemistry and Immunochemistry

Histochemistry: Cells were cultured into 12-well inserts plates until Day 18. After treatments (4 h, oleic acid or vehicle), they were fixed with Carnoy’s reagent (Sigma Aldrich), processed in paraffin, sectioned at 4 μm, stained with Alcian blue (AB, pH 2.5) followed by the periodic acid-Schiff reaction (PAS), and then counterstained with hematoxylin [43]. The AB/PAS method yielded blue color when mostly acidic mucins were present, purple when neutral mucins were also present and magenta when mainly neutral mucins were present. The corresponding cells were called stained mucus cells.

Immunochemistry: Inserts were removed, fixed in 90% ethanol for 24 h at −20 °C and processed into paraffin. Paraffin sections (4 μm) were rehydrated and endogenous peroxidase activity was quenched with 20 min of incubation in 5% H_2_O_2_/PBS. After washing slides, antigen retrieval was carried out by heating sections in 0.01 M citrate buffer (pH 6.0) by microwaving. After incubation in 2.5% normal horse blocking serum (ImmPRESS™, Vector, Cliniscience, Nanterre, France), sections were incubated for 30 min at room temperature with a rabbit polyclonal anti-MUC2 antibody (1/250; Santa Cruz Biotechnology (Cliniscience). The immune reactions were then detected by incubating with a ready-to-use peroxidase-labeled secondary reagent, ImmPRESS™ (MP-7401 for rabbit antibodies or MP-7402 for mouse antibody, Vector, Cliniscience) (30 min, room temperature). Control experiments were performed simultaneously omitting the primary antibody or incubating with pre-immune rabbit serum [42].

### 4.7. Fluorescent Cell Lipid Storage Analysis

Imaging and fluorescence quantifications were performed on Cytation 3 cell imaging reader (Biotek Instrument Inc., Colmar, France) on either living cells maintained at 37 °C or cells fixed with formalin 10% (Sigma Aldrich). For intracellular lipid labeling, living cells were incubated with AdipoRed Assay reagent (Lonza France SARL, Levallois-Perret, France) according to standard procedures. After AdipoRed assay, cells were fixed with formalin, washed in phosphate buffer saline (PBS) then treated with 0.1% Triton ×100 and nuclei were labeled with Hoechst 33258 (1 μg/mL) according to standard procedure (Sigma Aldrich). Fluorescence intensities were measured at 350 nm (excitation)/461 nm (emission) for Hoechst 33258 and 535/572 for AdipoRed. Images and statistical analyses on 96-wells or on E-plates 96 wells after fixation were performed at either ×4 or ×20 magnification with identical acquisition parameters of fluorescence. The ratios of AdipoRed fluorescent intensities normalized to those of Hoechst were calculated for each well and were confirmed by image fluorescence counting of AdipoRed intensity and Hoechst particle counts on at least 3 areas (1 mm^2^) per well and imaging at fixed conditions for AdipoRed at magnifications ×4 or ×20 with filters 469/525 and 586/647, for AdipoRed and Hoechst 33258, respectively. Data are presented as mean values ±SEM with significant differences for Student’s *t*-test *p*-values > 0.05.

### 4.8. qRT-PCR Analysis of Gene Expression

Total RNA purifications and first strand cDNA synthesis from cell cultures were performed according to [15]. Real-time quantitative PCR (Reverse transcription polymerase chain reactions, RT-qPCR) were performed with Absolute™ QPCR SYBR^®^ Green ROX Mix (Abgene, Courtaboeuf, France) on a Nano LC Light Cycler (Roche Diagnosis). Hypoxanthine phosphoribosyltransferase 1 (*HPRT1*) and fatty acid binding protein 1 (*FABP1*) primers were previously described [13], others were pre-designed KiCqStart primers from Sigma Aldrich. Levels of target mRNAs were normalized to those of *HPRT1* and measured at least in triplicates. Data are presented as mean values ± SD and significant differences were obtained using Student’s *t*-test (*p* < 0.05).

## 5. Conclusions

In conclusion, the co-culture of Caco-2 cells/HT29-MTX (90–10%) had a down-regulated transcription level of specific markers of epithelial differentiation (*HES1*, *Hath1*, *Gfi1*) when compared to Caco-2 or HT29-MTX cells alone. This highlights the importance of cellular interactions in biological functions, including nutrient absorption. Furthermore, exposure of Caco-2 cells alone, or in co-culture with HT29-MTX cells, to increasing concentrations of oleic acid pointed out major differences on the transcriptional regulation of phenotype markers (*TFF3*), fatty acid receptor (*GPR120*), stress responsive gene (*XBP1*), intracellular fatty acid transporter (*FABP1*, *-3*, and *-4*) and lipid droplet proteins (*PLIN5*, *ABDH5*, *CIDEB*, *CIDEC*). Taken together, bio-informatic analyses of signaling pathways and experimental comparison of Caco-2 differentiated alone, or in presence of goblet-like cells, confirmed the hypothesis that oleic acid represents a major supplier for full reconversion of cancer cells into healthy intestinal cells [33]. Overall, these results suggest that the co-culture (Caco-2 cells + HT29-MTHX) is a more physiologic model than Caco-2 cells alone but requires the presence of fatty acids to establish an intestinal barrier phenotype.

## Figures and Tables

**Table 1:** Gene datasets used for intestinal phenotype analysis. Datasets were retrieved from Gene Ontology Datasets (GEO) at www. ncbi.nih.gov, raised from fold changes (FcLOG), or published at COPE http://www.copewithcytokines.de/cope.cg. The number of genes commonly found in the 7000 genes analyzed are indicated.

Gene Dataset Name	Gene Number	Dataset Analysis	GEO	Caco-2 Diff vs. Undiff	Caco-2 Diff vs. HT29-MTX	Caco Diff vs. Entero (ileal + Juj)	Colonocyte	Enterocyte
				Common Gene Frequency % (Number)				
Caco-2 differentiated vs. undifferentiated	579	Pellis et al., 2005	GSE2047		13.6 (79)	51.1 (296)	1.9 (11)	2.1 (12)
Caco-2 differentiated vs. HT29-MTX differentiated (3 weeks)	1371	−2 < FcLOG < 2	GSE30292	5.8 (79)		61.7 (846)	0.9 (12)	0.9 (13)
Caco-2 differentiated vs. (enterocyte ileal + jejunal)	6038	−2 < FcLOG < 2	GSE30292	4.9 (296)	14.0 (846)		0.4 (22)	0.4 (28)
Colonocyte	103	COPE (PubMed list)		10.7 (11)	11.7 (12)	21.4 (22)		48.5 (50)
Enterocyte	142	COPE (PubMed list)		8.5 (12)	9.2 (13)	19.7 (28)	35.2 (50)	

**Table 2:** Gene transcription analysis of Caco-2, HT29-MTX cells, and co-cultures by qRT-PCR. Fc: fold change (normalized to Hypoxanthine phosphoribosyltransferase 1 HPRT) observed in co-culture versus theoretical (90% Caco-2 + 10% HT29-MTX) or co-cultures treated with oleic acid 60 μM for 24 h (Fc OA). Bold-type characters: up-regulated, italics: down-regulated; nd: not detected. Data are presented as mean values ± SD (*n* = 3).

Gene Symbol	Caco-2	HT29-MTX	Caco-2/HT29-MTX	Fc vs. Theoric	Fc OA Caco-2/HT29-MTX
**Intestinal phenotype matkers**					
*ATOH1 (Hath1)*	1366 ± 30	17,481 ± 1590	*212 ± 29*	−14	**3.4 ± 0.2**
*GFi1*	4.20 ± 0.10	**56.0 ± 9.4**	*0.7 ± 0.1*	−14	**3.9 ± 0.1**
*HES1*	1260 ± 338	1697 ± 36	*527 ± 29*	−2	**1.6 ± 0.1**
*MUC2*	**17.4 ± 1.6**	1.4 ± 0.2	*2.0 ± 0.5*	−8	**2.4 ± 1.7**
*MUC5*	1540 ± 10	**30,661 ± 1314**	*619 ± 27*	−7	**4.2 ± 0.0**
*SOX9*	228 ± 10	489 ± 83	158 ± 20	−2	*−3.2 ± 0.0*
*TFF3*	580774 ± 2	485,507 ± 15,791	101,000 ± 882	−6	**4.3 ± 0.2**
**Fatty acid uptake & transport**					
*FAT/CD36*	0.05 ± 0.03	**1.32 ± 0.35**	0.05 ± 0.00	−3	1.0 ± 0.1
*FABP1*	**263,971 ± 115,167**	27 ± 2	*50,176 ± 4078*	−5	**4.9 ± 0.4**
*FABP3*	**191 ± 18**	3.2 ± 0.5	98 ± 8	−2	**2.0 ± 0.5**
*FABP4*	**4.05 ± 0.62**	0.69 ± 0.14	*0.15 ± 0.03*	−25	**26.7 ± 8.3**
*GRP120*	2844 ± 91	**9007 ± 473**	*312 ± 8*	−11	**5.4 ± 0.2**
**Lipid droplet structure**					
*ABDH5 (CGI-58)*	**507 ± 158**	165 ± 14	*184 ± 28*	−3	**3.7 ± 1.5**
*PLIN1*	nd	**0.03 ± 0.004**	0.002 ± 0.001		−1.6 ± 0.1
*PLIN2 (ADFP)*	nd	nd	nd		
*PLIN3 (TIP47)*	**3199 ± 870**	1670 ± 63	*1200 ± 271*	−3	**2.7 ± 0.1**
*PLIN5 (oxPAT)*	0.005 ± 0.001	**0.031 ± 0.008**	0.001 ± 0.000	−10	**1.9 ± 0.1**
*CIDEA*	nd	nd	nd		
*CIDEB*	16.1 ± 2.8	18.1 ± 2.0	*3.1 ± 0.1*	−5	**3.2 ± 0.2**
*CIDEC*	**16,762 ± 2219**	4837 ± 64	*2217 ± 49*	−7	**2.3 ± 0.2**
**UPR stress response**					
*XBP1*	19,033 ± 634	61,810 ± 2713	13,454 ± 6654	−2	−1.4 ± 0.0
**Cancer marker**					
*TSPAN8*	124,836 ± 3825	706,666 ± 5070	19,088 ± 3500	−10	**4.7 ± 0.0**

## Supplementary Materials

Supplementary materials can be found at www.mdpi.com/1422-0067/18/7/1573/s1.

## Abbreviations

AB	Alcian Blue
ABDH5	α/β-hydrolase domain-containing protein 5
AMPK	Protein kinase AMP-activated
APOA2	Apoprotein 2
APOA3	Apoprotein 3
ATOH1 (Hath1)	Helix-loop-helix (HLH) protein
BMP4	Bone Morphogenic Protein 4
BSA	Bovine serum albumin
CIDEB and CIDEC	Cell-death activators CIDE-B and C
DGAT2	Diacylglycerol transferase 2
EGF	Epidermal growth Factor
FABPs	Fatty acid binding proteins
FASN	Fatty acid synthase
FAT/CD36	Fatty acid translocase
Gfi1	Growth factor independence 1
GPR120	G-protein coupled receptor 120
HES1	Hairy and enhancer of split-1
HPRT1	Hypoxanthine phosphoribosyltransferase 1
Klf4	Kruppel-like factor 4
LDLR	Low density lipid receptor
LIPE	Lipid protein lipase
MUC2	Mucin 2
MUC5AC	Mucin 5AC
OA	Oleic acid
PAS	Periodic acid-Schiff reaction
PLIN1, 3, 5	Perilipins 1, 3 and 5
PPARs	Peroxisome proliferator activated receptors (alpha, beta/delta and gamma)
qRT-PCR	Quantitative real-time polymerase chain reaction
RTCA	Real-time Cell Analyzer
SOX9	SRY-Box 9
SPDEF	SAM Pointed Domain Containing ETS Transcription Factor
TFF3	Trefoil fator 3
TSPAN8	Tetraspanin 8
XBP1	X-box binding protein 1
