# Supplementary material for: Oleic Acid Uptake Reveals the Rescued Enterocyte Phenotype of Colon Cancer Caco-2 by HT29-MTX Cells in Co-Culture Mode
Source: Int J Mol Sci. 2017 Jul 20;18(7):1573. doi: 10.3390/ijms18071573 (PMC5536061; doi:10.3390/ijms18071573)

		aFATCD36	SEM
APC/H	HT29-MTX	0.00288371	0.00057624
	Caco-2	0.00222729	0.00041429
	Co-culture	0	0.00028929
	Co-culture°	0.00229293	0.00043048

		aFABP4	SEM
PE/H	HT29-MTX	0.111468319	0.000655618
	Caco-2	0.069481626	0.001302511
	Co-culture	0.058280562	0.000533889
	Co-culture°	0.073680295	0.001237822

Figure S1: Extracellular FAT/CD36 and FABP4 proteins are reduced in Caco-2 cells co-cultured with HT29-MTX cells (10%). Differentiated cells (D20) were labeled with either anti-Allophycocyanin (APC) anti-FAT/CD36 antibody (1 µg/mL, Biolegend, Ozyme, Montigny-le-Bretonneux, France) without cell permeabilization or anti-FABP4 antibody (2 µg/mL,Sigma Aldrich) detected with phycoerythrin (PE)-coupled secondary antibody after permeabilization in triton 0,1%. Fluorescence intensity and quantification were measured on Cytation 3 plateform. Data are presented as mean fold changes (antibody fluorescence intensity / nuclei labeled with Hoechst 33258) +/- SEM on a representative experiment (n= 8 wells), stars represent significant Student t-test p-values p<0,05 between experimental *versus* theorical(°) (90% Caco + 10% HT29) co-culture results.

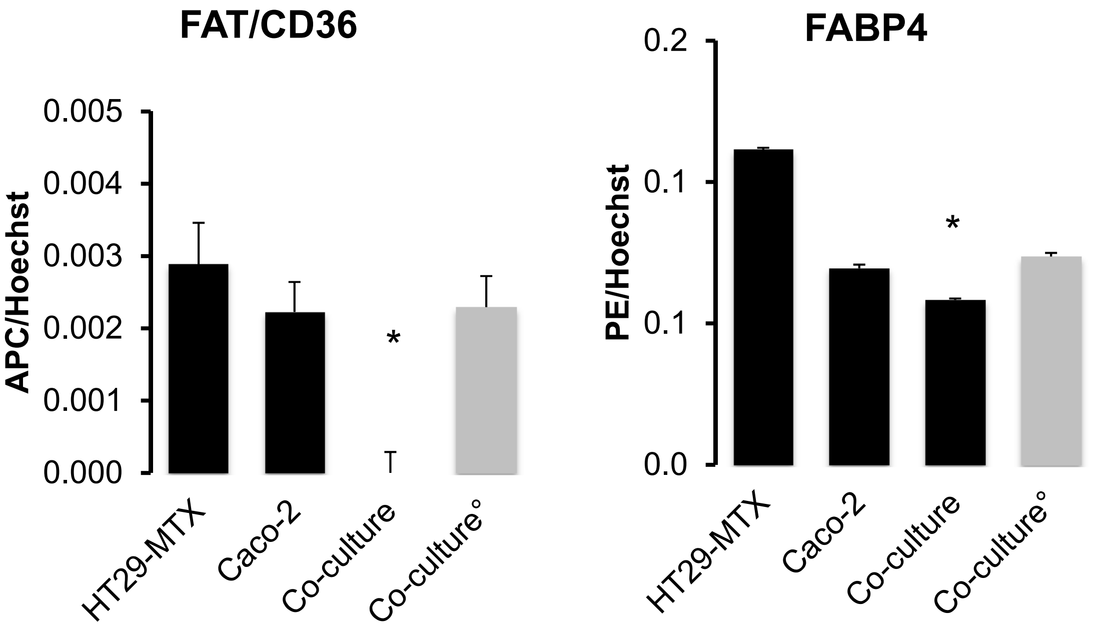

Supplement: Supplementary file 1 [file ijms-18-01573-s001.zip › ijms-202960-FS1-for final.pdf]
