# Supplementary material for: Oleic Acid Uptake Reveals the Rescued Enterocyte Phenotype of Colon Cancer Caco-2 by HT29-MTX Cells in Co-Culture Mode
Source: Int J Mol Sci. 2017 Jul 20;18(7):1573. doi: 10.3390/ijms18071573 (PMC5536061; doi:10.3390/ijms18071573)
Supplement: Supplementary file 1 [file ijms-18-01573-s001.zip › ijms-202960-Table S2.pdf]

Table S1: List of fatty acid regulatable genes related to human intestinal phenotype.

UNIQID	Name	GeneID	Cancer Biomarker	Caco Diff vs Undiff	Caco vs HT29 Diff	Caco diff vs entero ileal + jejunal	Colonocyte	Enterocyte
ACLY	ATP citrate lyase	47	x			x		
ACOX1	acyl-Coenzyme A oxidase 1, palmitoyl	119				x		
ADFP (PLIN2)	perilipin-2	131395		x				
ADIPOR2	adiponectin receptor 2	79602				x		
AFP	alpha-fetoprotein	174		x	x	x		
APOA2	apolipoprotein A-II	336		x	x	x		
APOC3	apolipoprotein C-III	345		x	x	x		
ATF3	activating transcription factor 3	467		x	x	x		
BHLHB2 (BHLHE40)	basic helix-loop-helix family, member e40	6280356				x		
CD36	CD36 molecule (thrombospondin receptor)	948	x			x		x
CEBPA	CCAAT/enhancer binding protein (C/EBP), alpha	1050				x		
CLDN4	claudin 4	1364				x		
CYP1A1	cytochrome P450, family 1, subfamily A, polypeptide 1	1543		x	x			
ESR1	estrogen receptor 1	2099	x	x				
FABP1	fatty acid binding protein 1, liver	2168	x	x	x			
FABP2	fatty acid binding protein 2, intestinal	2169		x		x		
FABP3	fatty acid binding protein 3, muscle and heart	2170		x	x			
FABP4 (AP2)	fatty acid binding protein 4, adipocyte	2167	x	x				
FABP5	fatty acid binding protein 5 (psoriasis-associated)	2171	x			x		
FASN	fatty acid synthase	2194	x	x		x		
FN1	fibronectin 1	2335	x	x	x	x		
G0S2	G0/G1 switch 2	50486				x		
GADD45a	growth arrest and DNA-damage-inducible, alpha	1647	x	x			x	
ID1	inhibitor of DNA binding 1, dominant negative helix-loop-helix protein	3397	x		x			
ID2	inhibitor of DNA binding 2, dominant negative helix-loop-helix protein	3398	x		x	x		

IDI1	isopentenyl-diphosphate delta isomerase 1	3422						
INSIG1	insulin induced gene 1	3638		x		x		
IRS1	insulin receptor substrate 1	3667				x		
LDHA	lactate dehydrogenase A	3939	x			x		
LDLR	low density lipoprotein receptor	3949				x		
LEP	leptin	3952	x				x	x
LIPC	lipase, hepatic	3990			x	x		
MUC3A	mucin 3A, cell surface associated	4584				x		
NFE2L2 (NRF2)	nuclear factor, erythroid 2-like 2	4780				x		
PGK1	phosphoglycerate kinase 1	5230			x	x		
PPARA	peroxisome proliferator-activated receptor alpha	5465		x				
PPARG	peroxisome proliferator-activated receptor gamma	5468		x		x		
PRKAR1A	protein kinase, cAMP-dependent, regulatory, type I, alpha	5573				x		
RBP4	retinol binding protein 4, plasma	5950	x		x	x		
SLC2A1 (GLUT1)	solute carrier family 2 (facilitated glucose transporter), member 1	6513	x	x		x		
SLC2A4 (GLUT4)	solute carrier family 2 (facilitated glucose transporter), member 4	6517		x				
TF	transferrin	7018			x	x	x	
TNFA	tumor necrosis factor	167910		x			x	
TSPAN8	tetraspanin 8	7103	x			x		
