# Supplementary material for: Oleic Acid Uptake Reveals the Rescued Enterocyte Phenotype of Colon Cancer Caco-2 by HT29-MTX Cells in Co-Culture Mode
Source: Int J Mol Sci. 2017 Jul 20;18(7):1573. doi: 10.3390/ijms18071573 (PMC5536061; doi:10.3390/ijms18071573)
Supplement: Supplementary file 1 [file ijms-18-01573-s001.zip › ijms-202960-Table S1.pdf]

Table S1: List of human genes specifically expressed in enterocytes versus colonocytes.

Symbol	Name	GeneID
A20	Tumor necrosis factor, alpha-induced protein 3 (TNFAIP3)	164371
ANP	Inhibin, beta A (INHBA)	1856335
AREG	amphiregulin	374
BDK	bradykinin	589
bFGF	Fibroblast growth factor 2 (basic) (FGF2)	177026
CCR1	chemokine (C-C motif) receptor 1	1230
CD11b (ITGAM)	Integrin, alpha M (complement component 3 receptor 3 subunit)	158221
CD11c (CCR4)	Chemokine (C-C motif) receptor 4 (CCR4)	159632
CD13 (ANPEP)	Alanyl (membrane) aminopeptidase	130949
CD14	CD14 molecule (CD14)	156684
CD151	CD151 molecule (Raph blood group)	977
CD18	Integrin, beta 2 (complement component 3 receptor 3 and 4 subunit) (ITGB2)	201337
CD21	Complement component (3d/Epstein Barr virus) receptor 2	240794
CD23	Fc fragment of IgE, low affinity II, receptor for (CD23) (FCER2)	680937
CD26	dipeptidyl-peptidase 4	197782
CD27	CD27 molecule	939
CD28	CD28 molecule	940
CD29	Integrin, beta 1 (fibronectin receptor, beta polypeptide, antigen CD29 includes MDF2, MSK12) (ITGB1)	2224984
CD31 (PECAM1)	Platelet/endothelial cell adhesion molecule 1	905159
CD32 (FCGR2A)	Fc fragment of IgG, low affinity IIa, receptor (CD32)	194554
CD35 (CR1)	Complement component (3b/4b) receptor 1 (Knops blood group)	190181
CD36	CD36 molecule (thrombospondin receptor)	948
CD39 (ENTPD1)	Ectonucleoside triphosphate diphosphohydrolase 1	1849599
CD41a	integrin subunit alpha 2b	3674
CD43 (SPN)	Sialophorin	2139082
CD44v6	CD44 molecule (Indian blood group) (CD44)	717487
CD49a	ITGA1 integrin subunit alpha 1	3672
CD49b	Integrin, alpha 2 (CD49B, alpha 2 subunit of VLA-2 receptor) (ITGA2)	697236
CD49c	Integrin, alpha 3 (antigen CD49C, alpha 3 subunit of VLA-3 receptor) (ITGA3)	172175
CD49f	Integrin, alpha 6 (ITGA6)	149927
CD59	CD59 molecule, complement regulatory protein	966
CD61 (ITGB3)	Integrin, beta 3 (platelet glycoprotein IIIa, antigen CD61)	165073

CD63	CD63 molecule	967
CD64	Fc fragment of IgG, high affinity Ia, receptor (CD64)	140094
CD66	Carcinoembryonic antigen-related cell adhesion molecule 1 (biliary glycoprotein) (CEACAM1)	903429
CD9	CD9 molecule	928
CSF3	colony stimulating factor 3 (granulocyte)	1440
DMBT1	deleted in malignant brain tumors 1	1755
EAAC1	Solute carrier family 1 (neuronal/epithelial high affinity glutamate transporter, system Xag), member 1	239952
FGF-15	Fibroblast growth factor 19 (FGF19)	169252
FIZZ-2	Resistin like beta (RETNLB)	182689
FPR1	formyl peptide receptor 1	2357
FPRL1	Formyl peptide receptor 2 (FPR2)	142766
FPRL2	Formyl peptide receptor 3 (FPR3)	240503
GITR	Tumor necrosis factor (ligand) superfamily, member 18 (TNFSF18)	169086
GRP	gastrin-releasing peptide	2922
HAI-1	SPINT1 serine peptidase inhibitor, Kunitz type 1	6692
HAI-2	SPINT2 serine peptidase inhibitor, Kunitz type 2	10653
hsp70	Heat shock 70kDa protein 4	141344
hsp90	Heat shock protein 90kDa alpha (cytosolic), class B member 1 (HSP90AB1)	509736
IDE	insulin-degrading enzyme	3416
IFRD1	interferon-related developmental regulator 1	3475
IGFBP1	insulin-like growth factor binding protein 1	3484
IL1RA	Interleukin 1 receptor, type I (IL1R1)	3093867
IL2	interleukin 2	3558
IL7	interleukin 7	3574
INHBA	inhibin, beta A	3624
INSR	insulin receptor	3643
IRAKM	Interleukin-1 receptor-associated kinase 3 (IRAK3)	197978
LBP	lipopolysaccharide binding protein	3929
LCRF	Diazepam binding inhibitor (GABA receptor modulator, acyl-CoA binding protein) (DBI)	140274
LGALS1	lectin, galactoside-binding, soluble, 1	3956
LGALS3	lectin, galactoside-binding, soluble, 3	3958
LTF	lactotransferrin	4057
MEP1A	meprin A, alpha (PABA peptide hydrolase)	4224
MEP1B	meprin A, beta	4225

MMP-1	Matrix metallopeptidase 1 (interstitial collagenase) (MMP1)	140712
MMP-10	Matrix metallopeptidase 10 (stromelysin 2) (MMP10)	131195
MMP-19	Matrix metallopeptidase 19 (MMP19)	2060044
MMP-26	Matrix metallopeptidase 26 (MMP26)	163051
NEU	V-erb-b2 erythroblastic leukemia viral oncogene homolog 2, neuro/glioblastoma derived oncogene ho	241389
NPW	neuropeptide W	283869
PAWR	PRKC, apoptosis, WT1, regulator	5074
PTHR	Parathyroid hormone-like hormone (PTHLH)	2060170
PYY	peptide YY	5697
REG3G	regenerating islet-derived 3 gamma	130120
S100G	S100 calcium binding protein G	795
S100P	S100 calcium binding protein P	6286
SIGIRR	single immunoglobulin and toll-interleukin 1 receptor (TIR) domain	59307
TIMP2	TIMP metallopeptidase inhibitor 2	7077
TIMP3	TIMP metallopeptidase inhibitor 3	7078
TLR2	toll-like receptor 2	7097
TLR9	toll-like receptor 9	54106
TOLLIP	toll interacting protein	54472
TRAF6	TNF receptor-associated factor 6, E3 ubiquitin protein ligase	7189
VEGFA	vascular endothelial growth factor A	7422
